# Clinical initiatives linking Japanese and Swedish healthcare resources on cancer studies utilizing Biobank Repositories

**DOI:** 10.1186/s40169-014-0038-x

**Published:** 2014-11-22

**Authors:** Toshihide Nishimura, Takeshi Kawamura, Yutaka Sugihara, Yasuhiko Bando, Shigeru Sakamoto, Masaharu Nomura, Norihiko Ikeda, Tatsuo Ohira, Junichiro Fujimoto, Hiromasa Tojo, Takao Hamakubo, Tatsuhiko Kodama, Roland Andersson, Thomas E Fehniger, Harubumi Kato, György Marko-Varga

**Affiliations:** First Department of Surgery, Tokyo Medical University, 6-7-1 Nishishinjuku Shinjuku-ku, Tokyo, 160-0023 Japan; Laboratory for Systems Biology and Medicine, Research Center for Advanced Science and Technology, The University of Tokyo, 4-6-1, Komaba, Meguro-Ku, Tokyo, 153-8904 Japan; Clinical Protein Science & Imaging, Biomedical Center, Dept. of Biomedical Engineering, Lund University, Lund, BMC D13, 221 84 Sweden; Biosys Technology, Daihyaku Seimei Toritsudai Ekimae Bldg 5 F 13-18, Nakane 2, Meguro-ku, Tokyo, 152-0031 Japan; ThermoFisher Scientific, 3-9 Moriya-cho, Yokohama, Kanagawa-ku, 221-0022 Japan; National Medical Center for Children and Mothers Research Institute, 2-10-1 Okura Setagaya-ku, 157-8535 Tokyo, Japan; Dept. of Biophysics and Biochemistry, Osaka University Graduate School of Medicine, 2-2 Yamadaoka, Suita, 565-0871 Japan; Department of Surgery, Clinical Sciences Lund, Lund University, and Skåne University Hospital, Lund, Sweden, 221 84 Lund, Sweden; Center of Excellence in Biological and Medical mass spectrometry (CEBMMS), Lund, 221 84 Sweden

**Keywords:** Cancer diseases, Protein quantification, Proteomics, Mass spectrometry, MRM, Biobanking, HUPO

## Abstract

The Tokyo Medical University Hospital in Japan and the Lund University hospital in Sweden have recently initiated a research program with the objective to impact on patient treatment by clinical disease stage characterization (phenotyping), utilizing proteomics sequencing platforms. By sharing clinical experiences, patient treatment principles, and biobank strategies, our respective clinical teams in Japan and Sweden will aid in the development of predictive and drug related protein biomarkers.

Data from joint lung cancer studies are presented where protein expression from Neuro- Endocrine lung cancer (LCNEC) phenotype patients can be separated from Small cell- (SCLC) and Large Cell lung cancer (LCC) patients by deep sequencing and spectral counting analysis. LCNEC, a subtype of large cell carcinoma (LCC), is characterized by neuroendocrine differentiation that small cell lung carcinoma (SCLC) shares. Pre-therapeutic histological distinction between LCNEC and SCLC has so far been problematic, leading to adverse clinical outcome. An establishment of protein targets characteristic of LCNEC is quite helpful for decision of optimal therapeutic strategy by diagnosing individual patients. Proteoform annotation and clinical biobanking is part of the HUPO initiative (http://www.hupo.org) within chromosome 10 and chromosome 19 consortia.

**Electronic supplementary material:**

The online version of this article (doi:10.1186/s40169-014-0038-x) contains supplementary material, which is available to authorized users.

## TMU/LUH - A Joint clinical center effort

The Tokyo Medical University Hospital, a pioneer in lung cancer treatment and surgery forms a center effort jointly with Lund University Hospital to build an expert capability resource. This joint establishment will intensify the utility of cancer expertise and experiences in both Japan and Sweden to benefit cancer patients. Clinical samples will be sampled from the hospitals with dedicated quality protocols and standard operating procedures (SOPs) by automated processing. These samples will be archived in Biobank storages, and will be built as a resource for R&D studies [1].

Today we have a lack of protein biomarker-, and imaging diagnostics within most cancer disorders. New clinical tools are expected to be used as early indicators of disease, or, as personalized indicator assays for targeted and stratified disease phenotype drug treatments in the near future. There is also a poor understanding of the mode of drug action mechanisms, by commonly used therapies, which is also true for new drugs introduced to the market. The actual targeted cells-, and proteins within disease, and the actual drug interactions are by no means understood for most medicines used in today’s therapies. These drug characteristics are needed for both efficacy-, and safety improvements, and also requested by regulatory authorities like; FDA/EMA/MHLW.

New technology developments that can be used to target the specificity and efficacy of new drug treatments within the health care system is progressing with increasing successes, in both Japan, North America and Europe. The new generation of drugs with mono-specific affinity will require more in depth knowledge in order to provide a tailor made cure to all patients taking the drug.

The aim and purpose of the Swedish-Japanese research program is to develop new insights into disease presentation and the disease progression. These objectives are intimately linked to the classification of patients that presents similar pathologies in disease. The stratification of these patients is an added value to both the healthcare sector as well as the drug industry, as it will point to the ability to optimize the treatment of cancer patients. A cornerstone of the Japanese-Swedish hospital initiatives will be to develop new Multiple Reaction Monitoring (MRM) Multiplex analysis methods for cancer, that can be used in combination with CT-imaging and pathophysiology diagnosis. At present, there is a lack of disease specific radiographic markers or protein/peptide biomarkers. These new biomarkers should be introduced into routine clinical practice to support clinical decision making for the management of common diseases that are consequent to life styles, but also to genetic heritage. The developed methods and technologies outlined here will bring new opportunities for describing the indices of pathogenesis that are associated with the processes of early disease development. Our research teams have previously been successful in presenting lung cancer biomarker candidates [2]–[4]. We propose to continue developing new Protein Biomarker Diagnosis Panels that can measure novel patterns of structural and functional marker expression that precede and predict certain disease development. The biology change within disease will be followed by alterations due to drug intervention. Drug compound localization in pulmonary tissue will be characterized in lung cancer and COPD tissue by imaging mass spectrometry. The aim is to further probe the drug and metabolite(s) content and distribution in tissues. Following administration and drug exposure, drug parent ion (*m/z*) and fragmented daughter ions will be analyzed by spatially localization, after scanning the histology section in the MALDI instrument at single cell level [5],[6]. The joint teams will also focus on excellence in biological and medical mass spectrometry, that provides health care institutions with expertise in clinical proteomics, drug imaging and mass spectrometry aiming at developing new diagnostic tools that can be integrated in routine clinical medicine. We have a major challenge in modern healthcare in both Japan and Sweden, to provide the right medicine to the right patient at the right time. Targeted treatment defined as “Personalized medicine” is becoming the next generation of drugs where drug efficacy and patient safety, is expected to predict the stratified treatment. In these developments, it is expected that patients will benefit by effective curing, and society will benefit by financial resources with increased efficiencies at a lower cost.

## Global healthcare and drug developments

There is a highly unmet need within the healthcare sector that calls for an increasing world-wide demand for new medicines and curative therapies to aid in the treatment of cancer patients.

We need to utilize and apply cutting edge research with state-of-art facilities more efficiently and drive opportunities that can be used in order to impact on providing quality care that extend prognosis.

In this respect, the pharmaceutical industry and its drug product provider responsibility is under major restructuring. The main objective for pharmaceutical industry is to provide more specific and more efficacious drugs by targeted treatment, i.e. by Personalized Medicine (PM), and to provide the diagnostic test that will direct the right patient group to the right drug. We outlined the directives of these principles recently in a white paper [7], predicting a future link in-between PM and diagnostic guidance. Today, this is an accepted concept and is being applied in Japan as the first country; The Clinical Practice Guideline, (from The Japan Lung Cancer Society (https://www.haigan.gr.jp/modules/guideline/index.php?content_id=3)).

In practice this means and results in that the Biomarker diagnostic research is increasing. Companion diagnostics is an area of impressive growth, where the pharmaceutical industry, the biotech sector, and academia are investing considerable resources.

**Figure 1:**
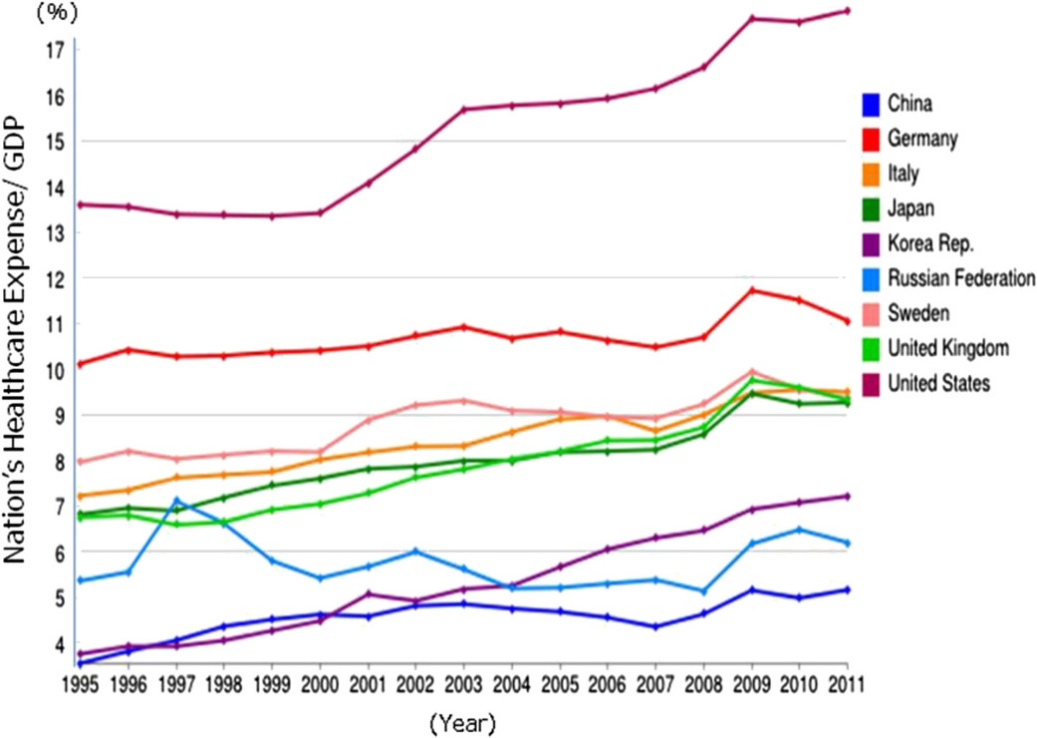
**Statistical presentation of national healthcare spending/GDP in countries in-between 1995-2012.**

The national healthcare expenses are also increasing for most countries in the western world, where the US is in the lead, spending >17% national healthcare expenses/GDP (Shown in Figure 1). The growth is almost 5% over the last two decades. Japan reaches almost 10% which is very similar levels of healthcare budgets as Sweden. The Japanese healthcare expense was 38.6 trillion yen in 2011 and 40 trillion yen in 2013. This cost continues to increase by 1 trillion yen/year, looking at the recent three years. Interestingly, China and Russia are on the lower end of healthcare spending with 5% and 6%, respectively.

The expenses that each country needs to provide for their respective healthcare is tightly associated with the development of new medicines.

Currently, the global pharmaceutical industry is worth $300 Bill, and is expected to be rising to $400 Bill by 2015. In addition, the market size for pharmaceuticals now exceeds $1 trillion and is continuously growing. This industry is very profitable, and currently employing about 1.5 million people within the US and EU-countries.

In this regards, the R&D investments in within this industry have consistently managed to grow with investments made within the biomedical sciences, having a total spend on R&D with more than $100 Bill, per annum.

As the Research and Development phases of drug development is a long-term investment for the pharma industry, taking into account that most drugs fail long before getting to market, there is an incentive strive to find new strategies and operating principles.

### Cancer research

Cancer diseases are some of the most costly for our societies. Processes essential to growth and replication of tumor cells and the maintenance of their supportive microenvironment such as the angiogenesis are today target strategies for cancer therapy.

Established chemotherapeutic agents and many novel agents in developments are directed towards various aspects of these essential cellular processes.

Over the years, we have been involved in developments associated with target proteins, possessing key regulatory functions in cancer [3],[8],[9]. They are as follows;

Growth factor signaling such as EGFR

VEGFR1, VEGFR2 and VEGFR3

RAS-RAF pathway

ERK-pathway

FGFR

PDGFR

BRAF

TGFB

As these growth factors stimulate cell growth by interacting with internal domains of the trans-membrane receptors, we will sequence the variety of targeted proteins in order to get a better understanding of the underlying tumor mechanisms. Previous data has been guiding in this respect [10],[11].

Other oncological processes that will be directed within the joint cancer studies incorporate;

- metastasis
- angiogenesis that includes VEGF
- DNA replication, including transcription and repair
- histone acetylation and de-acetylation that can be mapped by mass spectrometry

Upon growth factor receptor binding to targets such as EGF, FGF, and TGFB, the activation of tumor biology is initiated [3],[5],[6],[8],[12]. This ligand-receptor complex formation also imposes conformational changes of the receptor which will result in an activation of the transfer of phosphate groups. The autophosphorylation loop of the kinase is the target area of mass spectrometry deep-mining of these regions within the protein sequence [13]–[15].

Establishing the identity of the amino acids that are phosphorylated is of high interest.

By dedicated clinical study materials from various patient groupings our objective is to map the receptor-ligand interactions. The glycolytic post-translational-modifications (PTM) of any given ligand activator will be directly linked to the binding affinity. The PTM status of the receptor itself has also proven to be of major importance to the signaling mechanisms [16]. We recently outlined a strategic overview of key protein targets, playing a key role in pancreatic cancer [16]. The TGF-β receptor and TGF- β ligand has a direct role in the development of lung cancer as well as other cancer forms [15].

On a molecular level, the intracellular targets, with specific assigned function in cancer are enzymes which activities catalysis the signaling cascade through the cellular regions reaching the nucleus. We have previously been working with ERK and MAPKAP signaling, as well as ZAP70. ZAP70 is a key target in a number of cancer types and is responsible for the autophosphorylation, of specific threonine positions within the protein that can be determined [13],[14]. The stoichiometric distribution of phosphor-groups being post-translationally modified has been pioneered by our research team. Sequencing of the target peptide of this tyrosine kinase can be accomplished by both ESI- as well as MALDI- mass spectrometry.

We recently reported on the development of biomarkers on the newly identified neuroendocrine phenotype within lung cancer [4], also denamed “The diffuse neuroendocrine system”.

**Figure 2:**
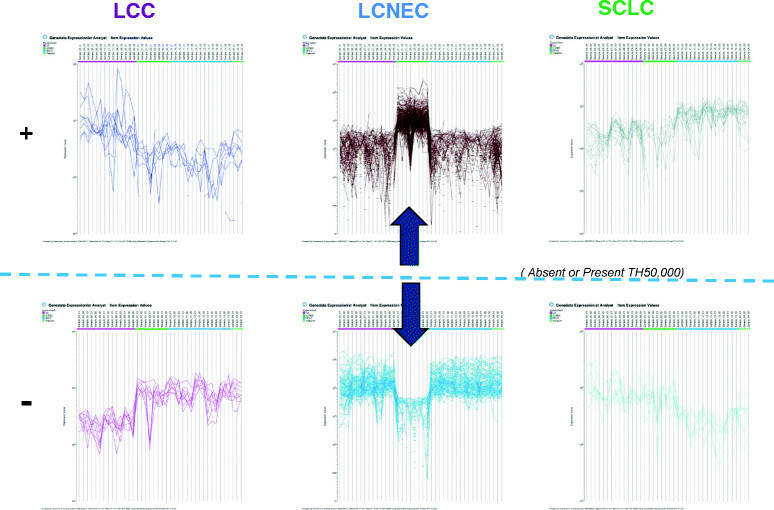
**Deep sequencing of proteins in LCNEC patient samples in comparison to LCC and SCLC.**

We have followed up with new protein sequencing experiments where the expression specificity of the LCNEC phenotype can be identified, as shown in Figure 2. A clear differential expression pattern can be seen in comparison to LCC and SMC. The difference in expression constitutes proteins with both medium-, as well as low-abundant expression (unpublished data).

Interestingly, these LCNEC Carcinoid tumors originate from cells that belong to the diffuse neuroendocrine system. These cells resembles nerve cells in certain ways, but they are also alike hormone-making endocrine cells in other ways. This phenotype of tumor cells are localized throughout the body, found in organs like the lungs, intestines, as well as the stomach [2].

### Protein deep sequencing platform and biobanking

Mining the data output from protein deep sequencing studies is an intense research activity where the chromosome initiative has presented impressive novel annotation deliveries [17]–[21]. The HUPO database; NextProt provides evidence detail on status output of chromosome 10 and 19 (http://www.nextprot.org).

Currently the remaining unknown proteins, coded by the human genome are 27% [17].

These are recent data generated from the Proteome Exchange database and include post-translational modifications as well as splice variants and protein isomers [22]. Details of the experimental outputs were published in a special issue in 2014, presenting the major developments from the C-HPP chromosome teams [18],[19],[21],[23]. 22 global chromosome team published papers on the novel annotations, including database reports with neXtProt, PeptideAtlas, and CAPER.

**Figure 3:**
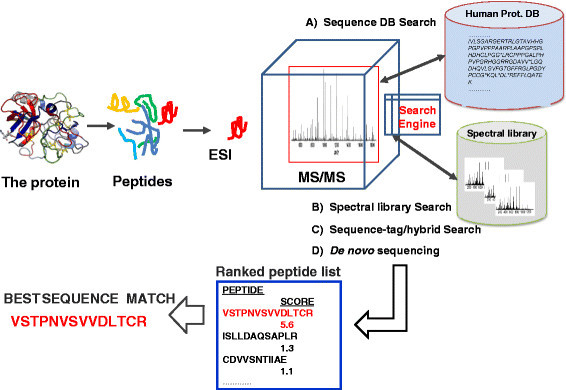
**Workflow with LC-MS platform integrated to search engines for the annotation of proteins.**

A recent development was made jointly by the TMU-LUH research teams, assigning a specific protein deep sequencing platform and work-flow. We are currently applying the process in lung cancer studies where we target novel proteoforms of missing gene coded proteins. The workflow process as we currently use in laboratories in both Tokyo and Lund is depicted in Figure 3.

**Figure 4:**
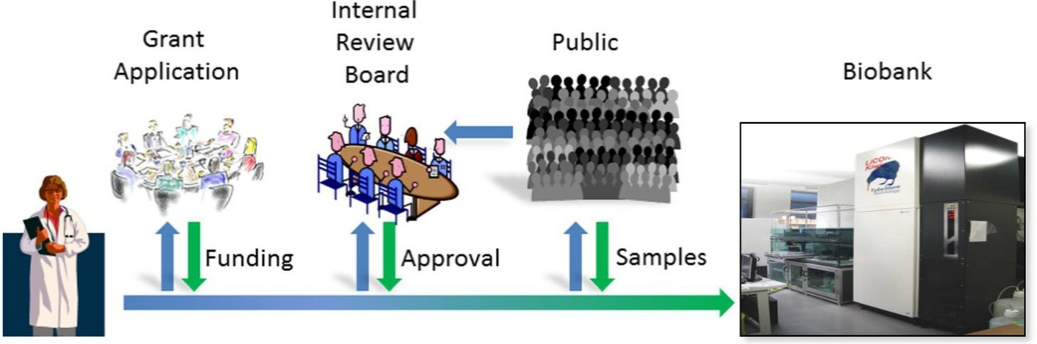
**Exploring new governance paradigms for Biobanks.**

Large scale Biobanking is presently an intense clinical activity in hospitals around the world. Recently, Biobanking was recognised by the TIME Magazine as One of; “10 Ideas Changing the World Right Now”. We will continue to establish biobank capabilitiesis, a first step towards realizing the vision of a comprehensive access to TMU-LUH biobank samples. We will combine these samples with health register data, thereby identifying correlations in-between clinical variables and disease presentations in patients. Currently we are using a resource driven sample collection, as outlined in Figure 4. We are coordinating sample collections in-between TMU and LUH, utilizing robotic -80 °C sample handling systems as previously described [1],[24]–[27]. The ultra-low temperature storage is essential as it ensures the stability of proteins within the clinical samples. Blood samples will be processed by robotic liquid handling and aliquoted in 384-sample tube systems, developed by our research team [25]. The 384 high-density rack system will provide a cost-benefit advantage, and expected to become a new standard in modern biobanking. This allows HUPO to easily process Biobank samples globally.

## Author’s contributions

All authors equally assisted in drafting the manuscript. All authors read and approved the final version.

## Author’s information

Professor Toshihide Nishimura and Professor György Marko-Varga are principal investigator for the Sweden-Japan research project. Assoc. Professor Takeshi Kawamura, Dr. Yutaka Sugihara, Dr. Yasuhiko Bando, and Dr. Shigeru Sakamoto are experts of mass spectrometry. Dr. Masaharu Nomura, Professor Norihiko Ikeda, Professor Tatsuo Ohira, Professor Junichiro Fujimoto, Professor Hiromasa Tojo, Professor Takao Hamakubo, Professor Tatsuhiko Kodama, Professor Junichiro Fujimoto, Professor Hiromasa Tojo are clinical scientist, Professor Roland Andersson is the vice dean and head of surgery, Professor Thomas E. Fehniger is a pathologist and director of CEBMMS. Harubumi Kato is a head clinician in Japan.

## Authors’ original submitted files for images

Below are the links to the authors’ original submitted files for images. Authors’ original file for figure 1 Authors’ original file for figure 2 Authors’ original file for figure 3 Authors’ original file for figure 4
