# Supplementary figures and images for: Clinical initiatives linking Japanese and Swedish healthcare resources on cancer studies utilizing Biobank Repositories
Source: Clin Transl Med. 2014 Nov 22;3:61. doi: 10.1186/s40169-014-0038-x (PMC4303744; doi:10.1186/s40169-014-0038-x)

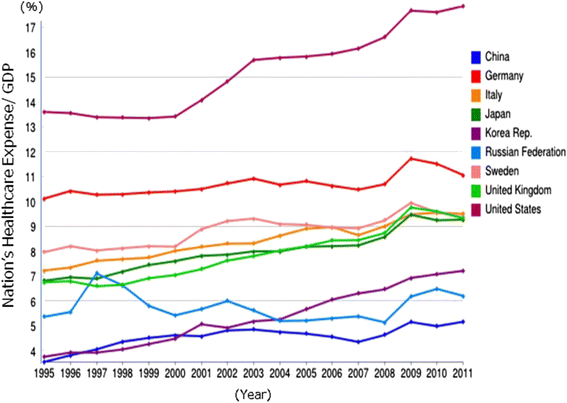

Supplement: Supplementary file 1 — Authors’ original file for figure 1 [file 40169_2014_38_MOESM1_ESM.gif]

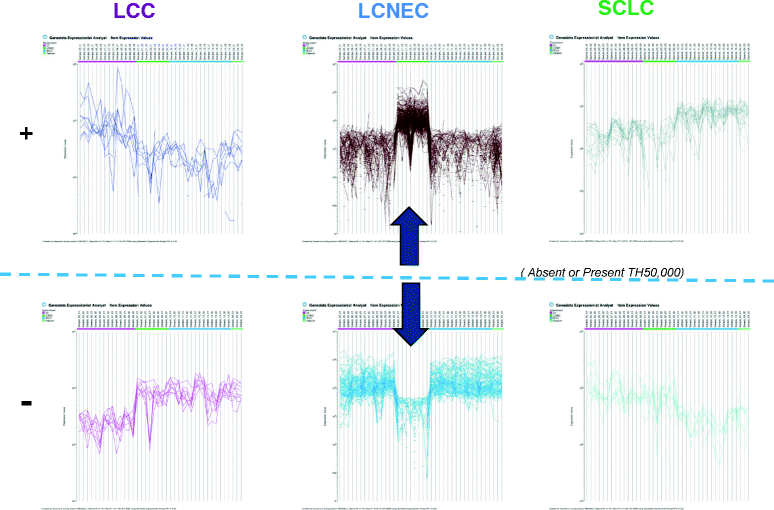

Supplement: Supplementary file 2 — Authors’ original file for figure 2 [file 40169_2014_38_MOESM2_ESM.gif]

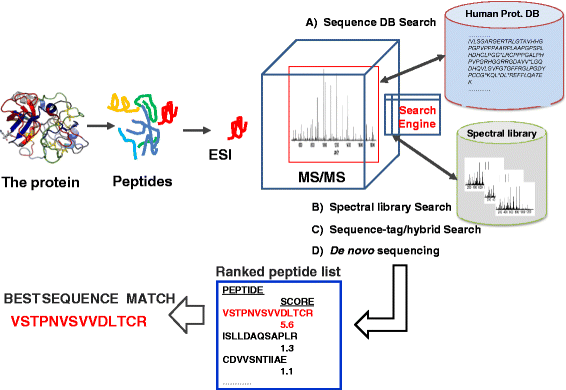

Supplement: Supplementary file 3 — Authors’ original file for figure 3 [file 40169_2014_38_MOESM3_ESM.gif]

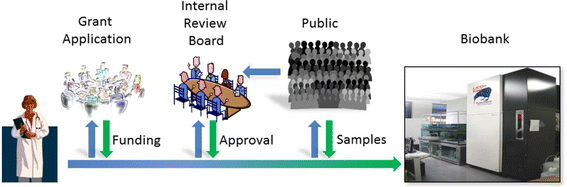

Supplement: Supplementary file 4 — Authors’ original file for figure 4 [file 40169_2014_38_MOESM4_ESM.gif]
